# Transplantation versus Long-Term Left- or Bi-Ventricular Assist Device Implantation: Major Challenges for Decision-Makers

**DOI:** 10.31083/j.rcm2309321

**Published:** 2022-09-16

**Authors:** Michael Dandel

**Affiliations:** ^1^German Centre for Cardiovascular Research (DZHK), Partner Site Berlin, 10785 Berlin, Germany

Shortly after the first heart transplantation (HTx) in December 1967, which ended with the early death of that patient, several HTx were performed at the Stanford University and Texas Medical Center. In 1968 the Texas group reported that only 7 of their 11 patients had a normal cardiac function up to 4.5 months after transplantation and, in 1971, only 27% of their 26 allograft recipients were alive at 2 years after HTx [1]. Given those poor results, elective HTx was suspended by that group after about 2 years [1, 2]. At the Stanford University, thanks to particularly intensive and continuous efforts made to improve allograft rejection surveillance (e.g., introduction in 1973 of the trans-venous endomyocardial biopsies [EMBs] into the clinical praxis), the initially discouraging HTx results had improved steadily and in 1974 that group reported an increase of the 2-year survival rate from 26% to 40% [1].

Despite the progresses made in surgery, it became increasingly evident that only major improvements of the immunosuppressive strategies and rejection surveillance can turn HTx into a reliable therapeutic option for end-stage heart failure (HF). Indeed, after the worldwide first incorporation of cyclosporine A (CsA) into the immunosuppressive regimens in 1980, plus the development in 1981 of a four-grade rejection severity classification to provide a more reliable basis for treatment, the 2-, and 3-year survival rates reported in 1985 by the Stanford group reached 75%, and 70%, respectively [1, 3, 4]. For HTx performed worldwide between 1982–1991, the overall median survival rate reached 8.6 years [5]. The immunosuppressant regimens underwent further optimization after 1991, especially by introduction of tacrolimus as an alternative treatment option to CsA and by the introduction of mycophenolate mofetil (instead of azathioprine) into the maintenance immunosuppressant therapy regimens in combination with either CsA or tacrolimus [6]. A study from Korea on 201 consecutive adults who underwent HTx between 1992-2008, revealed 1-, 5-, and 10-year survival rates of 95.5%, 86.9%, and 73.5%, respectively [7]. For patients transplanted worldwide between 2002–2009, the overall median survival rate increased up to 12.5 years [5]. The recognition of antibody-mediated rejection (AMR) as a distinct entity since 2005 contributed substantially to the further optimization of cardiac rejection surveillance and therapy [8]. Because no single invasive or non-invasive surveillance method can provide all the necessary information to achieve the goal of avoiding rejection-related damages of the allograft, most transplant centers use combined non-invasive and invasive surveillance strategies. Particularly useful for non-invasive surveillance of allograft function still remain more advanced echocardiographic techniques like tissue Doppler imaging (TDI) and speckle-tracking echocardiography (STE) which allow the replacement of routine EMBs by optimally timed of diagnostic EMBs and can help in therapeutic decision-making for patients with EMB-proven low grade histopathological and immuno-pathological acute rejection [9].

Despite the progresses made in HTx, their annual numbers began to level off already after 1990 due to the shortage of donor hearts and it became obvious that HTx cannot keep pace with the rising demand [1]. Although HTx remains the best treatment for terminal HF, only the alternative to implant a long-term left- or bi-ventricular assist device (LVAD or BiVAD) if, for different reasons, HTx is not or not yet feasible, can further improve the outcome of patients with drug-refractory end-stage HF. The initial use of LVADs only as a bridge to transplantation (BTT) was extended after 2000 also for a permanent ventricular support (destination therapy [DT]) [10]. With the increasing use of continuous flow LVADs, the 2-year survival of LVAD recipients increased from 31% up to 70–78%, and recently, the 5- and 7-year survival rate reached 54% and 51%, respectively [10].

**Fig. 1.**
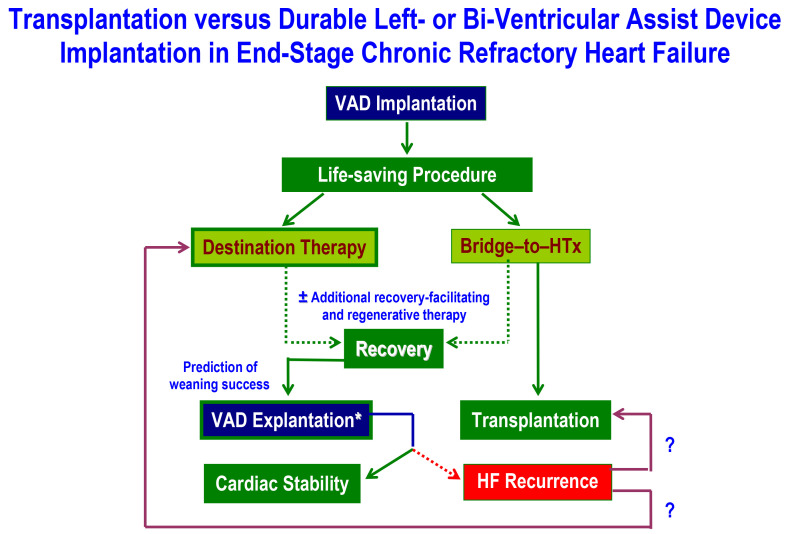
**Interconnections between long-term mechanical ventricular support and heart transplantation in the management of patients with end-stage heart failure**. VAD, ventricular assist device; HF, heart failure. * explantation can be successful even after incomplete cardiac recovery [11]. ? indicates that in case of HF recurrence, there are these 2 alternatives (i.e., transplantation or implantation of a second VAD). Dotted arrows indicate lower probability.

Already 27 years ago it was observed that, in some patients with drug-refractory chronic non-ischemic cardiomyopathy (NICMP), a BTT can turn into a bridge-to-recovery (BTR) allowing device explantation. Later, with the increasing use of VADs also as DT, it was proved that even DT-VADs may become a BTR (Fig. 1, Ref. [11]). The worldwide first elective explantations of long-term LVADs in patients with idiopathic dilated cardiomyopathy were performed in Berlin where already between 1995 and 1999, 23 patients were weaned from their LVADs [12]. Until 2015, a total of 116 adults were weaned in Berlin (59 of them with pre-implant NICMP, considered previously irreversible), after evidence of relevant and stable ventricular reverse remodeling and functional recovery [12]. A long-term evaluation of 53 weaned patients with end-stage NICMP as the underlying cause for VAD implantation, revealed 5- and 10-year post-explant survival probabilities (including post-HTx survival for those with HF recurrence) of 72.8% and 67%, respectively [11]. Post-weaning 5- and 10-year survival probabilities only from HF recurrence or weaning-related complications reached in those patients 87.8% and 82.6%, respectively [11].

Timely detection of HF patients at high risk for sudden cardiac worsening leading to dependency on permanent inotrope infusions, and finally to the need for HTx or VAD support is crucial for effective management of advanced HF. This presupposes complex multidisciplinary investigations and integrative interpretation of a large variety of clinical, hemodynamic, imaging (including STE) and laboratory data, which alone would be unable to predict short-term patient outcome [13, 14].

In LVAD candidates echocardiography is required in decision-making regarding the need for an additional temporary right ventricular (RV) support or maybe a BiVAD [15]. Echocardiography is also required for intraoperative guiding of VAD implantation, postoperative surveillance and optimization of the VAD support, monitoring of the RV in LVAD recipients, and search for adverse circumstances which can impair VAD therapy. Echocardiography has decisively contributed to the key finding that prolonged LVAD support can trigger and further promote myocardial reverse remodeling and improvement of ventricular function up to levels which allow LVAD explantation. It still remains the major tool for detection and estimation of VAD-promoted myocardial remission or even recovery, plus prediction of long-term cardiac stability without VAD support, with a key role in selection of weaning candidates and weaning decision-making [12].

Given the complexity of the theoretical and practical challenges related to HTx and long-term VAD support, further research is needed to improve and optimize these vital therapies for drug-refractory end-stage HF. Assessment of the myocardial recovery potentials in LVAD candidates and search for additional recovery-facilitating therapies during LVAD support deserve also particular attention.
